# Shape-stabilized phase change material with highly thermal conductive matrix developed by one-step pyrolysis method

**DOI:** 10.1038/s41598-021-80964-8

**Published:** 2021-01-12

**Authors:** Shibin Wu, Yan Chen, Zhenshou Chen, Jiaqi Wang, Miaomiao Cai, Junkai Gao

**Affiliations:** School of Naval Architecture and Maritime, Zhejiang Ocean University, Zhoushan, 316022 China

**Keywords:** Energy storage, Renewable energy

## Abstract

Metal microspheres doping porous carbon (MMPC), which was prepared using in-situ pyrolysis reduction strategy, could enhance the thermal conductivity of shape-stabilized phase change material (ss-PCM) prepared by MMPC as the matrix. However, in previous studies that were reported, the preparation of MMPC needed to synthesize porous carbon by pyrolysis firstly, and then porous carbon adsorbed metal ions was pyrolyzed again to obtain MMPC, which was tedious and energy-prodigal. In this study, a one-step pyrolysis strategy was developed for the synthesis of MMPC through the pyrolyzation of wheat bran adsorbed copper ions, and the copper microspheres doping wheat bran biochar (CMS-WBB) was prepared. The CMS-WBB was taken as the supporter of stearic acid (SA) to synthesize the ss-PCM of SA/CMS-WBB. The study results about the thermal properties of SA/CMS-WBB demonstrated that the introduction of copper microspheres could not only improve the thermal conductivity of SA/CMS-WBB, but also could increase the SA loading amount of wheat bran biochar. More importantly, the CMS-WBB could be obtained by only one-step pyrolysis, which greatly simplified the preparation process and saved energy consumption. Furthermore, the raw material of wheat bran is a kind of agricultural waste, which is abundant, cheap and easy to obtain. Hence, the SA/CMS-WBB synthesized in this study had huge potentialities in thermal management applications, and a simplified method for improving the thermal properties of ss-PCMs was provided.

## Introduction

Energy storage exerts an extraordinary impact on balancing the energy supply and demand^1^. Phase change materials (PCMs) has received considerable attention in energy area, because they could absorb or release plentiful heat in the process of phase change^2^. PCMs can be classified to be organic, inorganic and organic–inorganic mixture phase change material^3^, and among them, the organic PCMs such as fatty acids, alcohols, paraffin, etc., have the advantages of favorable chemical stability, high phase transition enthalpy, low supercooling and no phase separation^4–6^. Among the organic PCMs, stearic acid (SA), a kind of fatty acid, has the advantages of high heat capacity, suitable melting temperature and small volume change during phase transition process. However, stearic acid still has the disadvantages of leakage and low thermal conductivity when it is directly used as phase change material^7–9^. To overcome the liquid leakage and low thermal conductive defects of organic PCMs, the shape-stable phase change materials (ss-PCMs) were prepared using the porous matrixes as the supports of organic PCMs^10,11^. There are many types of matrixes were used to prepare ss-PCMs, including mesoporous carbon^12^, meso-porous silica^11^, graphene^13^, carbon nanotubes^14^, metal foams^5^, etc.

Among these carriers, biochar attracted much attention owing to its large specific surface and abundant functional groups, which was prepared by pyrolysing biomass, such as forestry and agricultural residues^15^. For example, Chen et al. used almond shell biochar (ASB) as supporting material to load polyethylene glycol (PEG) for the synthesis of PEG/ASB ss-PCMs, and the experimental results showed that PEG/ASB had excellent thermal stability and phase transition properties^16^. Although the leakage problem of PCMs could be solved by using biochar as carrier, the thermal conductivity of ss-PCMs still needed to be further improved. For example, Wan et al. incorporated palmitic acid into pinecone biochar to synthesize a new type of ss-PCMs, and its thermal conductivity was only 0.3926 W/mk^17^. Atinafu et al. immobilized 1-hexadecyl alcohol with cotton porous carbon, and the composite material has 0.41 W/mK thermal conductivity^18^. To facilitate the improvement of the thermal conductivity of ss-PCMs based on biochar, metal particles, such as metal nanoparticles^19^, metal microparticles^20^, were added into the ss-PCMs. However, after several phase transformation cycles, the metal materials were easy to agglomerate or precipitate because of the high density of the metal particles.

To improve the diffusion uniformity of metal particles in the ss-PCMs, our group used biochar to adsorb copper ions, and copper ions were reduced to copper microspheres by in-situ reduction method to obtain copper microspheres doped biochar. Then, ss-PCMs were prepared with copper microspheres doped biochar as the carrier of organic PCMs. The results showed that this method could make the thermal conductivity of PCMs improved successfully^21^. The in-situ reduction method did not need extra reductant, and copper microspheres could be firmly fixed on the surface of biochar, which effectively improved the dispersion stability of copper microspheres during the phase change process of ss-PCMs. Nonetheless, this method needed to firstly prepare biochar by pyrolysis, and then utilized biochar to adsorb copper ions. After that, the biochar adsorbed with copper ions was pyrolized, and the biochar doping with copper microspheres was obtained. The above method developed by our group still had the problems of complicated process and high energy consumption. Therefore, a more simple method which is based on in-situ reduction and used to increase the thermal conductivity of ss-PCMs is needed.

**Figure 1:**
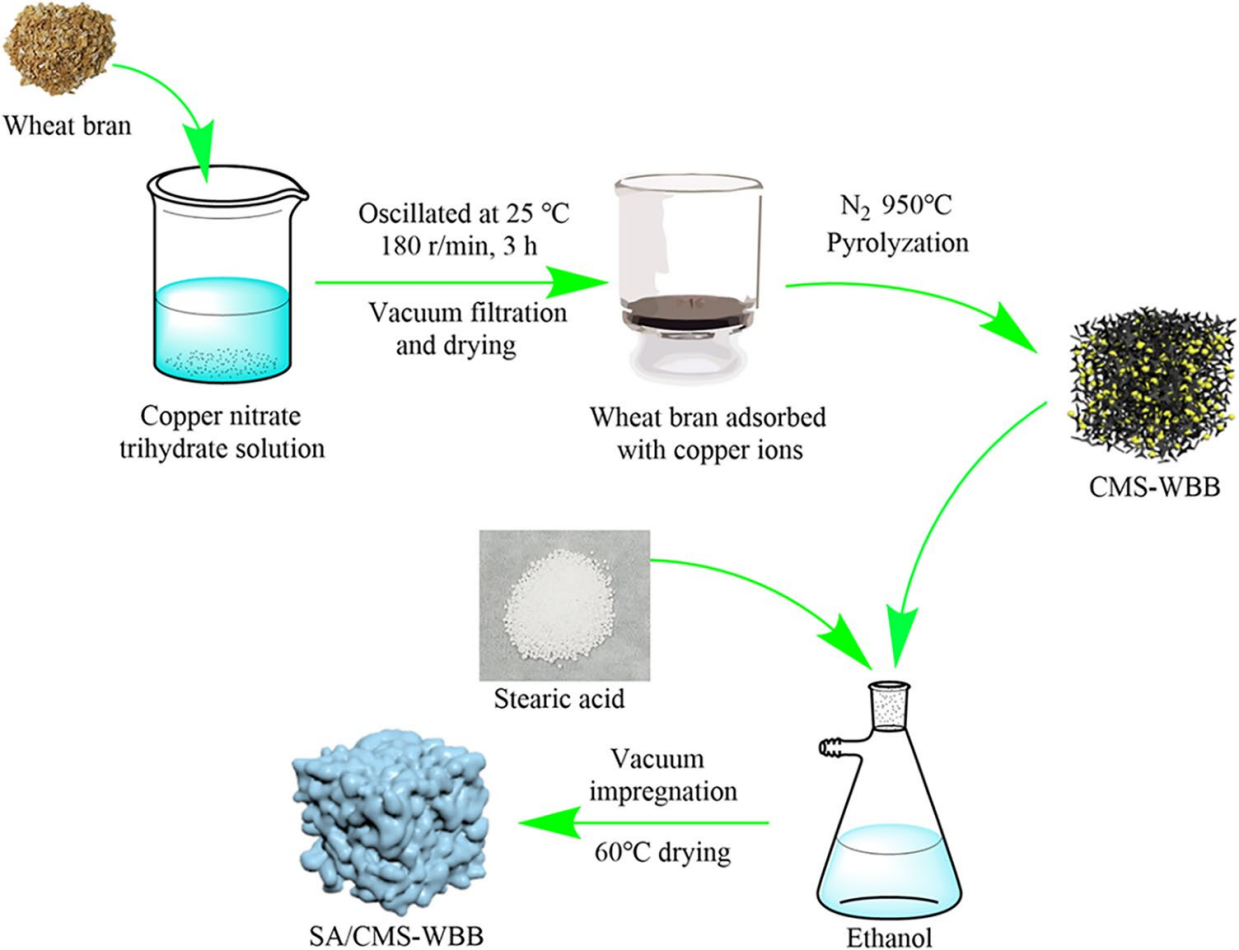
Schematic diagram of SA/CMS-WBB synthesization.

Enlightened by direct adsorption of metal ions by biomass, in this study, the wheat bran was used as raw material to adsorb copper ions directly, and then the wheat bran containing copper ions was pyrolyzed in a quartz tube furnace in nitrogen atmosphere. In this process, the wheat bran was pyrolyzed to be wheat bran biochar (WBB), and the copper ions were reduced to be copper microspheres (CMS), and therefore, the wheat bran biochar doping with copper microspheres (CMS-WBB) could be obtained by only one step, which greatly simplified the preparation process and saved energy consumption. Then, the CMS-WBB was used as the carrier of SA, and a new type of ss-PCMs (SA/CMS-WBB) was prepared, and the specific synthesis process of SA/CMS-WBB can be seen in Fig. 1. More importantly, it was found that the introduction of copper microspheres could improve the thermal conductivity of SA/CMS-WBB and increase the SA loading amount of wheat bran biochar.

Therefore, in this paper, the effects and influence mechanism of the introduction of copper microspheres on the SA loading amount and thermal conductivity of SA/CMS-WBB were studied in detail. The morphology and pore structure of CMS-WBB were characterized, and meanwhile, the crystalline and thermal storage properties of SA/CMS-WBB were investigated. The results demonstrated that SA/CMS-WBB could be regarded as a favorable material used in the aspect of energy storage, and thus, in this paper a more convenient approach to improve the thermal property of ss-PCMs was provided.

## Experimental

### Materials and methods

Wheat bran was obtained from Hulin City in the Heilongjiang province of China. Stearic acid was supplied by Aladdin Biochemical Technology Co., Ltd. Copper nitrate trihydrate and ethanol were provided by Shanghai Chemical Reagent Company.

### Preparation of CMS-WBB

The wheat bran was cleaned by distilled water and then dried at 100 °C, and then ground into powder. Then, 1 g of wheat bran powder was added into 100 ml copper nitrate solution (1000 mg/L). The obtained mixed solution was put into a constant temperature oscillator and oscillated for 3 h at 25 °C, and then the resultant suspension was filtered, and the precipitate was dried in an oven at 50 °C for 12 h. Finally, the dried precipitate was heated to 950 °C with the heating rate of 3 °C/min in nitrogen atmosphere and pyrolyzed to prepare CMS-WBB.

### Preparation of SA/CMS-WBB

The SA/CMS-WBB was synthesized by vacuum impregnation method. Firstly, a predetermined amount of stearic acid was dispersed in absolute ethanol (20 ml). Then a certain amount CMS-WBB was added to the solution after the stearic acid was completely dissolved. After vacuum stirring for 1 h, the mixed solution was transferred to water-bath where the temperature was 60 °C and kept stirring until the ethanol completely evaporated. Finally, SA/CMS-WBB was obtained at the bottom of the vacuum filtration bottle in the form of black solid. The weight ratio of SA in the composite of SA/CMS-WBB was designed to be 30%, 40% and 50%, and the final product was named as SA/CMS-WBB-1, SA/CMS-WBB-2 and SA/CMS-WBB-3, respectively. In the control group, SA/WBB-1 and SA/WBB-2 were prepared by using pure WBB as carrier, and the weight ratios of SA in SA/WBB-1 and SA/WBB-2 were 30% and 40%, respectively.

### Characterization

The size and morphologies of WBB, CMS-WBB, SA/WBB and SA/CMS-WBB were observed by scanning electron microscopy (SEM, Quanta FEG-250). The characteristic functional groups of SA, WBB, CMS-WBB, SA/WBB and SA/CMS-WBB were recorded by Fourier transform infrared spectroscopy (FT-IR, Bruker VECTOR22, Karlsruhe, Germany) with a scanning range of 500–4000 cm^−1^. The crystal structure of the samples was detected by X-ray diffraction (XRD, DX-2700, SHL-2, Thermo Scientific) under certain condition (Voltage at 40 kV, Current at 40 mA, diffraction range of 10–60°). The changes of elements on the surface of WBB before and after adding copper microspheres were explored by X-ray photoelectron spectroscopy (XPS) device of ESCALAB 250Xi K-Alpha. The thermal stabilities of the SA, SA/WBB and SA/CMS-WBB samples were tested by thermogravimetric analysis (TGA, HCT-1, Beijing) with the protective gas of nitrogen, and the heating rate and temperature range were 10 °C/min and 50–500 °C respectively. The thermal properties of composite PCMs were evaluated by a NETZSCH DSC200-F3 thermal analyzer, with the shielding gas of nitrogen at the temperature range of 0–100 °C, and the heating and cooling rate was 10 °C/min. The pore structures of WBB and CMS-WBB were studied by nitrogen adsorption technique (Quanta, NOVA2000E, USA). The specific surface area of multiple points was determined by Brunauer–Emmett–Teller (BET) method, and the pore size distribution of desorption isotherm branches was obtained by Barrett-JoynerHalenda (BJH) model. The thermal constant analyzer (CTPS-2500, FRD, China) was utilized to test the thermal conductivities of WBB, SA/WBB and SA/CMS-WBB.

## Results and discussion

### Characterization of WBB, CMS-WBB and SA/CMS-WBB

**Figure 2:**
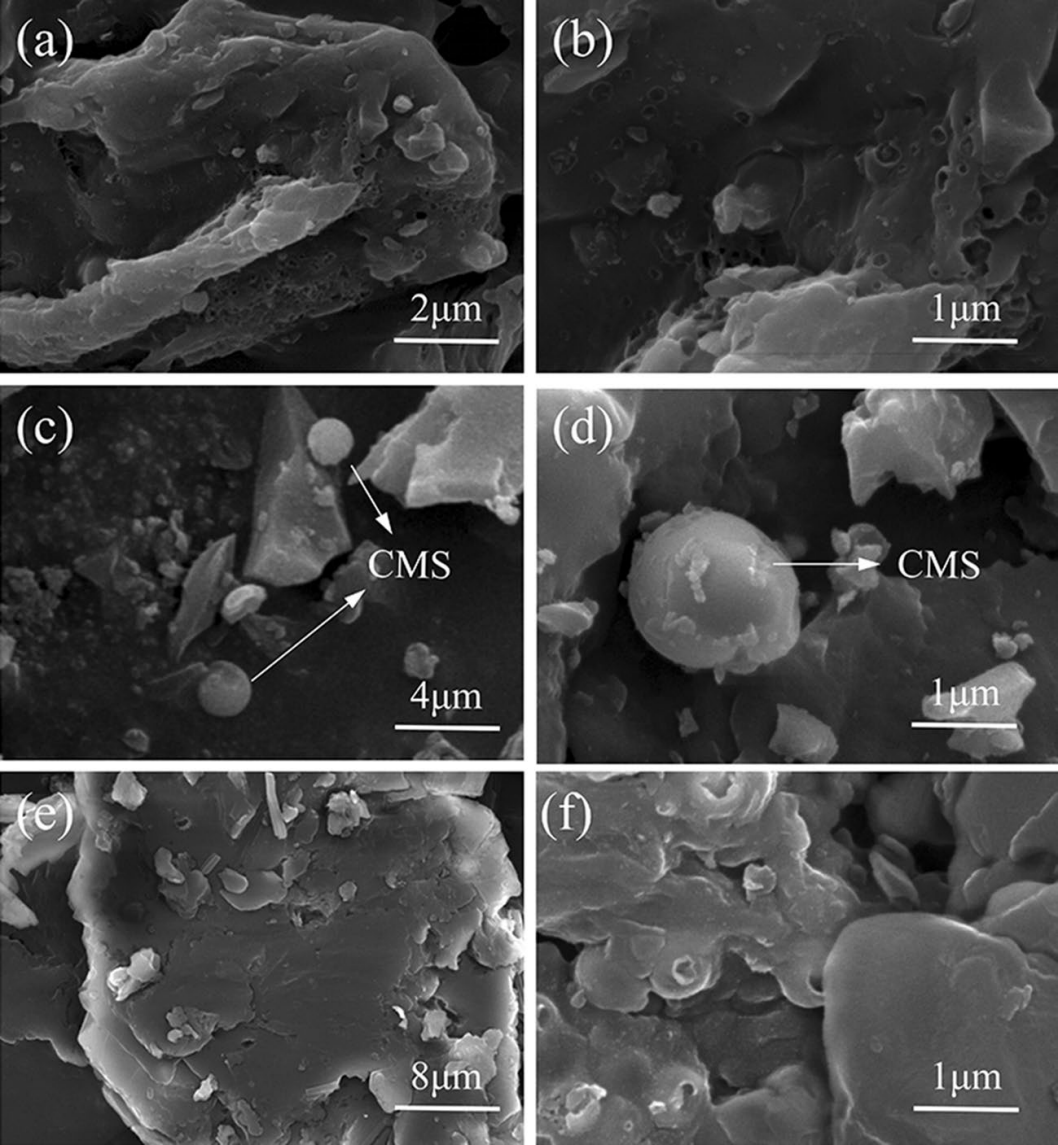
SEM images of WBB (**a**, **b**), CMS-WBB (**c**, **d**) and SA/CMS-WBB-2 (**e**, **f**).

The microscopic structures and surface morphology of WBB, CMS-WBB and SA/CMS-WBB-2 were studied by SEM. Figure 2 displays the SEM images of WBB, CMS-WBB and SA/CMS-WBB-2. As can be observed in Fig. 2(a,b) that the WBB was made up of biochar particles with rich pores, and when copper ions were reduced to copper microspheres, spherical copper particles could be seen on the surface of CMS, which could be seen in Fig. 2(c,d). The spherical copper particles were randomly distributed on the surface of WBB, and therefore, CMS was successfully attached to WBB. Moreover, Fig. 2(e,f) showed that the surface of SA/CMS-WBB-2 was relatively smooth because the hole and surface of CMS-WBB were covered with a layer of SA, which showed that SA was completely fixed by the carrier of CMS-WBB.

**Figure 3:**
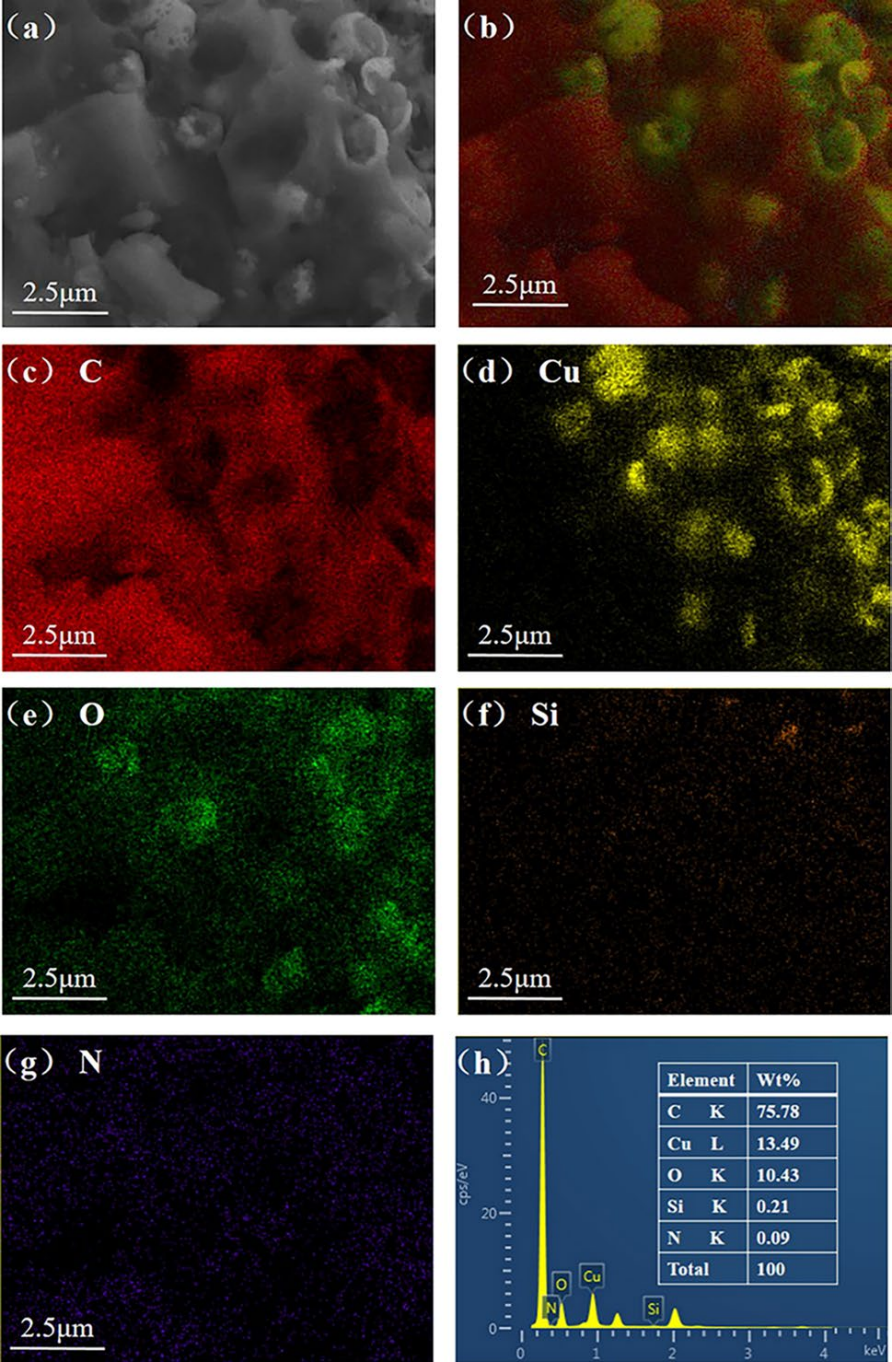
SEM image of CMS-WBB (**a**), EDS layered image of CMS-WBB (**b**), element mapping of C (**c**), Cu (**d**), O (**e**), Si (**f**) and N (**g**) on CMS-WBB, EDS spectra of CMS-WBB (**h**).

The EDS element mapping could be used to show the spatial distribution of the elements of CMS-WBB. As shown in Fig. 3, biochar was mainly composed of C. The spatial distribution map of C element preliminarily showed the size and shape of the pores on the surface of CMS-WBB. The spatial distribution pattern of Cu element clearly showed that the copper microspheres are successfully added to the biochar and unevenly distributed on the surface of the biochar. The spatial distribution of O element was similar to that of Cu element, which was due to the partial oxidation of copper particles in CMS-WBB. The trace distribution of Si was mainly derived from the residual SiO_2_ ash in the wheat bran powder before pyrolysis^22–24^.

### Pore properties of WBB and CMS-WBB

**Figure 4:**
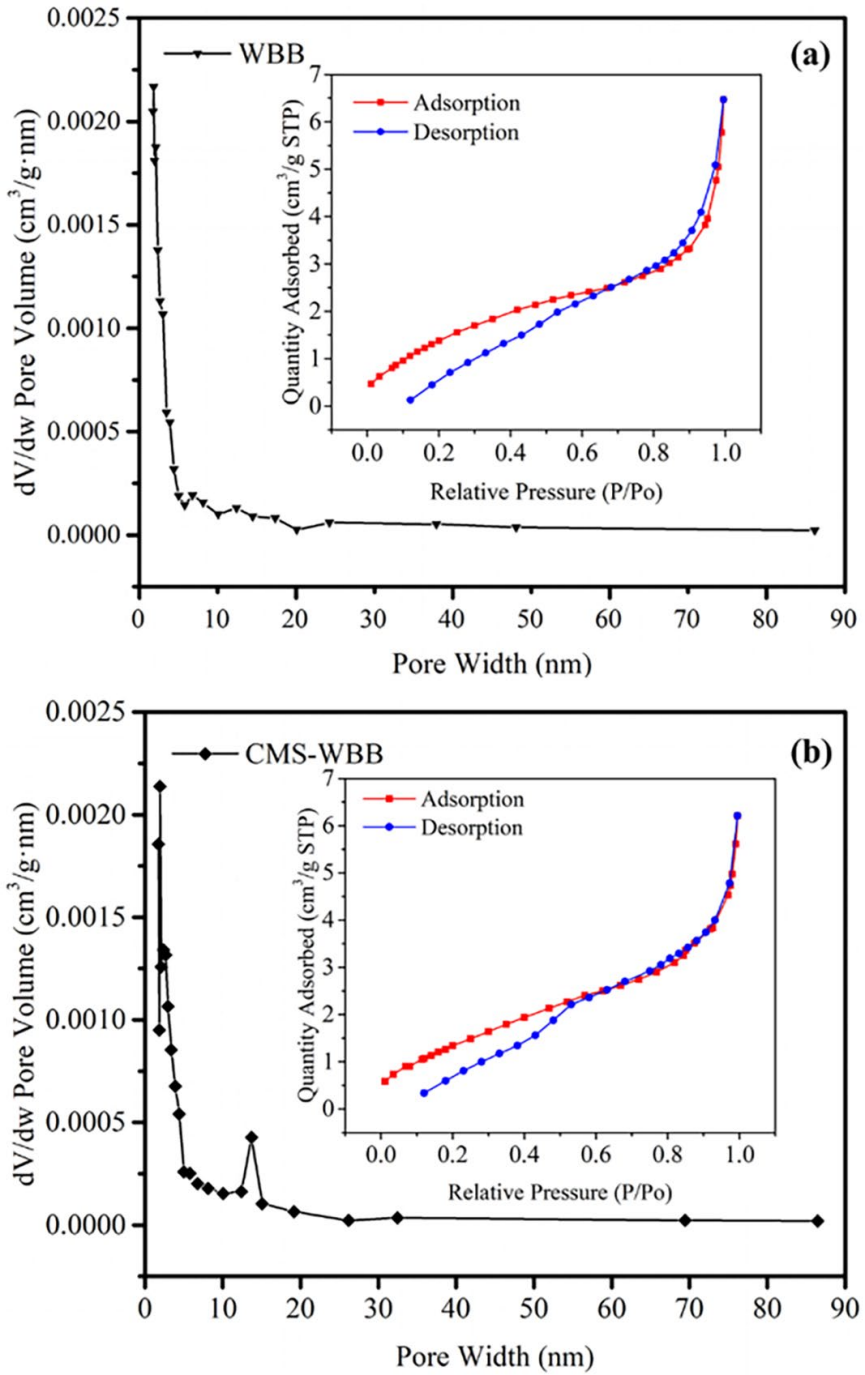
N_2_ adsorption and desorption isotherms and pore diameter distribution of WBB (**a**) and CMS-WBB (**b**).

The pore size distribution of WBB and CMS-WBB were evaluated by the Nitrogen adsorption–desorption method, and the results are illustrated in Fig. 4. The figure shows that the nitrogen adsorption–desorption isotherms of WBB and CMS-WBBD were type II/IV, and both of them had hysteresis loops^25^. The hysteresis loops were not closed, which might be owing to the fact that the pores of the two samples were small and dispersed, resulting in part of the nitrogen remaining in the pores of the samples during desorption, and this result was consistent with the pore width distribution curve^11,12^. The specific surface area and pore volume of WBB was obtained by BJH and BET calculation methods, which were 26.5278 m^2^/g and 0.008393 cm^3^/g, and the corresponding values of CMS-WBB were 29.0147 m^2^/g and 0.008216 cm^3^/g. It was clear that, CMS-WBB had larger specific surface area than WBB, which was beneficial to increase the loading capacity of CMS-WBB for organic phase change material.

### Leakage tests of SA, SA/CMS-WBB and SA/WBB

**Figure 5:**
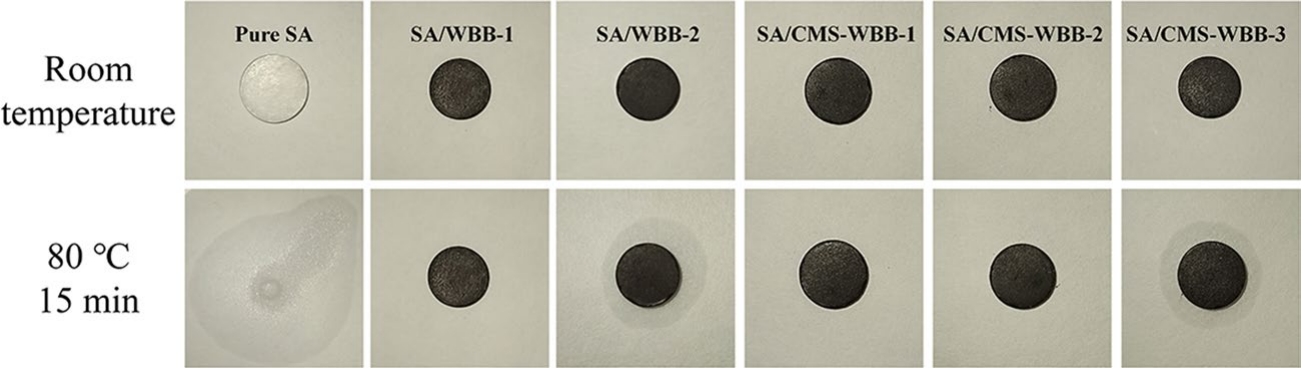
Leakage tests of SA, SA/CMS-WBB and SA/WBB.

In order to confirm whether the sample will leak at high temperature, SA, SA/CMS-WBB and SA/WBB were pressed into small wafers and put into an oven at 80 °C for 15 min. When the SA/CMS-WBB composite material leaked, SA stains were left at the bottom of the culture dish, and the results are displayed in Fig. 5. It was clear that pure SA was melted in large quantities, and SA/CMS-WBB-3 was slightly leaked. However, SA/CMS-WBB-1 and SA/CMS-WBB-2 were not leaked. The main reason was that the porous structure of CMS-WBB brought capillary effect and surface tension to SA molecules, so that SA could be fixed by CMS-WBB without leakage, and therefore, the largest proportion of stearic acid in CMS-WBB was 40%. Additionally, in the control group, the sample of SA/WBB-2 showed a slight leakage, and no leakage for SA/WBB-1 was observed. Therefore, the maximum loading amount of stearic acid in WBB was 30%. The results demonstrated that the loading capacity of CMS-WBB for SA was increased after doping with copper microspheres, which was attributed to the increase in specific surface area of CMS-WBB after the introduction of copper microspheres, and this result was consistent with that of N_2_ adsorption–desorption tests mentioned above.

### Chemical properties of SA, SA/CMS-WBB-1, WBB, CMS-WBB

**Figure 6:**
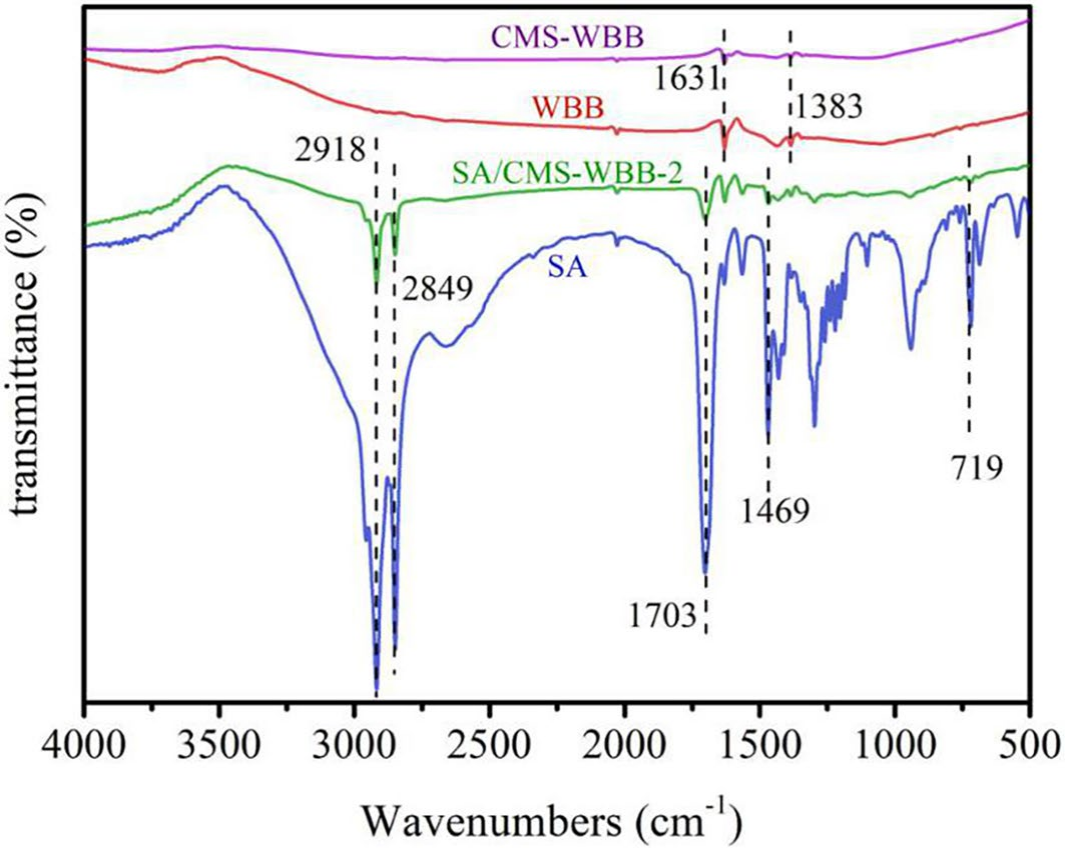
FTIR spectra of WBB, CMS-WBB, SA/CMS-WBB-2, and SA.

The FTIR spectra of WBB, CMS-WBB, SA/CMS-WBB-2, and SA are displayed in Fig. 6. It could be illustrated from the pattern of WBB and CMS-WBB that the positions of diffraction peaks were the same, and the shapes and sizes were similar. This result indicated that, the formation of copper microspheres on the surface of WBB did not change the superficial chemical functional groups of WBB. In the spectra of WBB and CMS-WBB, the characteristic absorption peak at 1631 cm^−1^ originated from the tensile vibration of C=C group, while the peak at 1383 cm^-1^ was caused by C–C^16,26,27^. In addition, there were wide peaks at 1010–1120 cm^−1^, which were typical graphite properties^18,28^. For the pure SA, the characteristic peaks are as follows: 2917 cm^−1^ and 2849 cm^−1^ were ascribed to C–H bond stretching vibration, 1703 cm^−1^ to the C=O stretching band, 1469 cm^−1^ to the C–O bending vibration, 719 cm^−1^ to the vibration of aliphatic chains of stearic acid^5,14,15^. Through comparing the patterns of CMS-WBB, SA, and SA/CMS-WBB-2, it could be found that the characteristic peaks of the SA were detected in the spectrum of SA/CMS-WBB-2, and there was no new peak emerged. Therefore, SA/CMS-WBB-2 did not undergo chemical changes during the synthesis process ^18,29,30^. Through the above analysis, it could be concluded that the composite PCMs synthesized in the study have favorable chemical stability.

### Crystallization ability of SA, SA/CMS-WBB-2, CMS-WBB, and WBB

**Figure 7:**
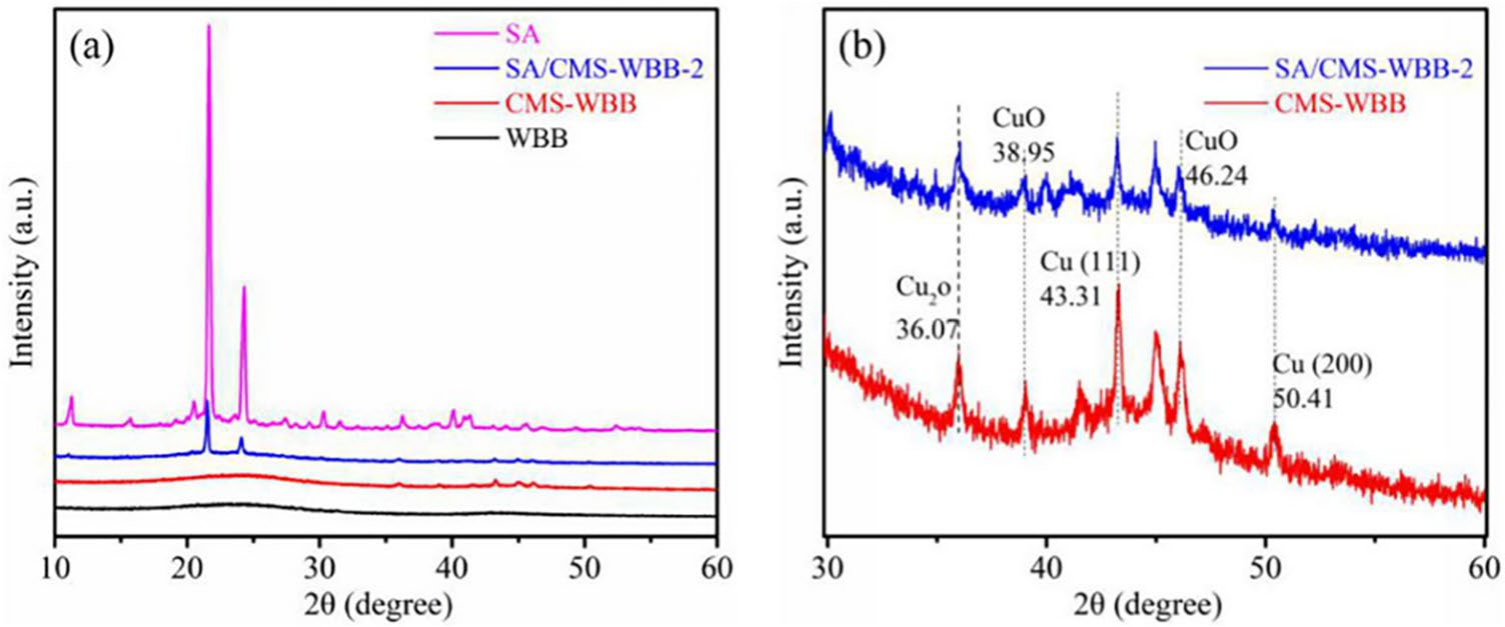
XRD patterns of SA, SA/CMS-WBB-2, CMS-WBB, WBB (**a**) and XRD patterns of SA/CMS-WBB-2 and CMS-WBB highlighted in range of 2θ = 30°–60° (**b**).

The XRD pattern of SA, SA/CMS-WBB-2, CMS-WBB, and WBB are shown in Fig. 7. The pattern of WBB had two broad peaks at 23.27° and 42.92°, and there was no obvious diffraction peak, indicating that WBB was essentially amorphous^31,32^. For CMS-WBB, which was formed by adding copper microspheres to WBB, there were two typical diffraction peaks at 43.31° and 50.41°, which represented the diffraction of (111) and (200) planes of cubic copper, and the same peaks also appeared in SA/CMS-WBB-2 synthesized by using CMS-WBB as carrier, which indicated that copper microspheres were successfully added to WBB^4,33^. In addition, the broad and weak peaks of WBB and CMS-WBB at 23.27° and 42.92° correspond to the (002) and (100) planes of the graphite crystal, which indicated that both WBB and CMS-WBB formed disordered graphite crystallite structure^23,34^. The XRD patterns of SA/CMS-WBB-2 and CMS-WBB were expanded in the 2θ = 30°–60°, as shown in Fig. 7(b). It could be found that there was a diffraction peak at 36.07°, which was caused by Cu_2_O, and the diffraction peaks at 38.95° and 46.24° belonged to CuO^35,36^. Therefore, in addition to copper nanoparticles, CuO and Cu_2_O also existed in CMS-WBB, which indicated that the copper particles were partially oxidized. For pure stearic acid, two typical diffraction peaks were observed at 21.63° and 24.29°, which showed that SA had abundant crystallinity. After SA was impregnated into CMS-WBB, the same diffraction peaks of SA and the broad peak of WBB could be observed from SA/CMS-WBB-2 in the same range, which suggested that the intercalation between the SA and CMS-WBB did not change the crystal structure of SA. There was no new diffraction peak in the pattern of SA/CMS-WBB-2 compared with that of SA, and therefore, there was no chemical reaction in the loading process^37–40^. In addition, the typical peak value of SA in SA/CMS-WBB-2 was less than that of pure SA, which was attributed to that the content of SA in the composite was reduced and the crystallinity of SA in SA/CMS-WBB-2 decreased under the limitation of the porous structure of CMS-WBB.

### Surface element analysis of WBB and CMS-WBB

**Figure 8:**
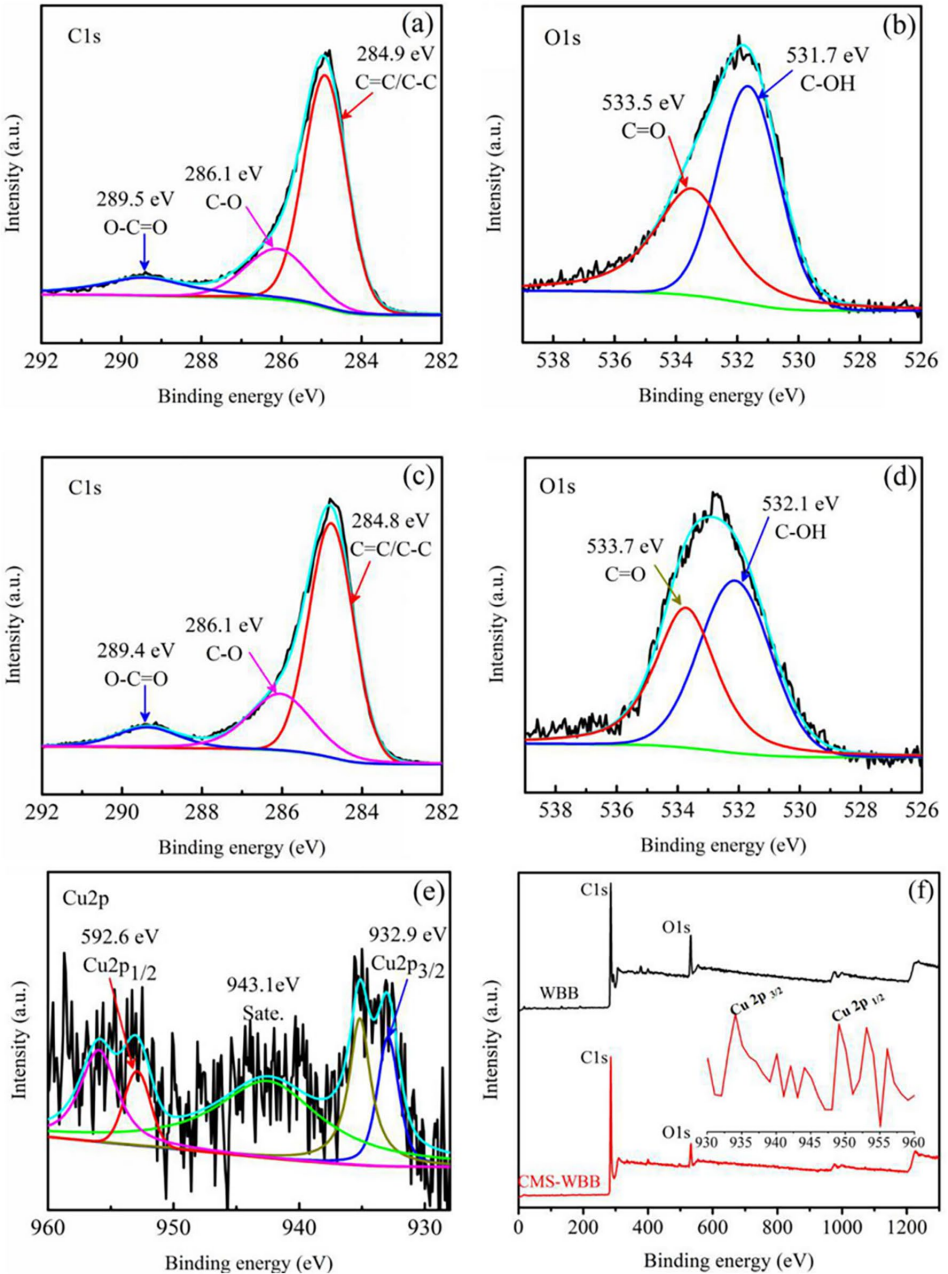
Photoelectron spectra of WBB: C1s (**a**), O1s (**b**); photoelectron spectra of CMS-WBB: C1s (**c**), O1s (**d**), Cu2p (**e**); full XPS spectrum of the WBB and CMS-WBB samples (**f**).

XPS was used to explore the changes of elements on the surface of WBB and CMS-WBB, and the results are shown in Fig. 8. The peaks of C1s and O1s in WBB were attributed to C=C/C–C (284.8 eV), C–O (286.1 eV), O–C=O (289.5 eV), C–OH (531.7 eV) and C=O (533.5 eV). The C1s and O1s peaks of CMS-WBB were assign to C=C/C–C (284.8 eV), C–O (286.1 eV), O–C=O (289.4 eV), C–OH (532.1 eV) and C=O (533.7 eV), which were basically the same as that of WBB^41–43^. Figure 8(e) shows the XPS spectrum of Cu2p. It could be clearly observed that the peak at 932.9 eV corresponded to Cu2p_3/2_ and the peak at 952.6 eV corresponded to Cu2p_1/2_, which indicated that the copper particles were successfully added to the biochar^33,44–46^. In addition, there was a shaking satellite peak at 943.1 eV between the two peaks. Compared with the smooth curve of pure copper between the two peaks, it was indicated that the copper particles in the CMS-WBB sample were partially oxidized^44,45^. According to the XPS patterns of WBB and CMS-WBB in Fig. 8(f), it could be found that in the curve of WBB, the peak intensity of O1s was higher than that of O1s in the curve of CMS-WBB, and the ratio between carbon and oxygen in CMS-WBB was reduced, which might be due to the reaction between the oxygen and copper during the one-step pyrolysis process.

### Thermal stability analysis of SA and SA/CMS-WBB-2

**Figure 9:**
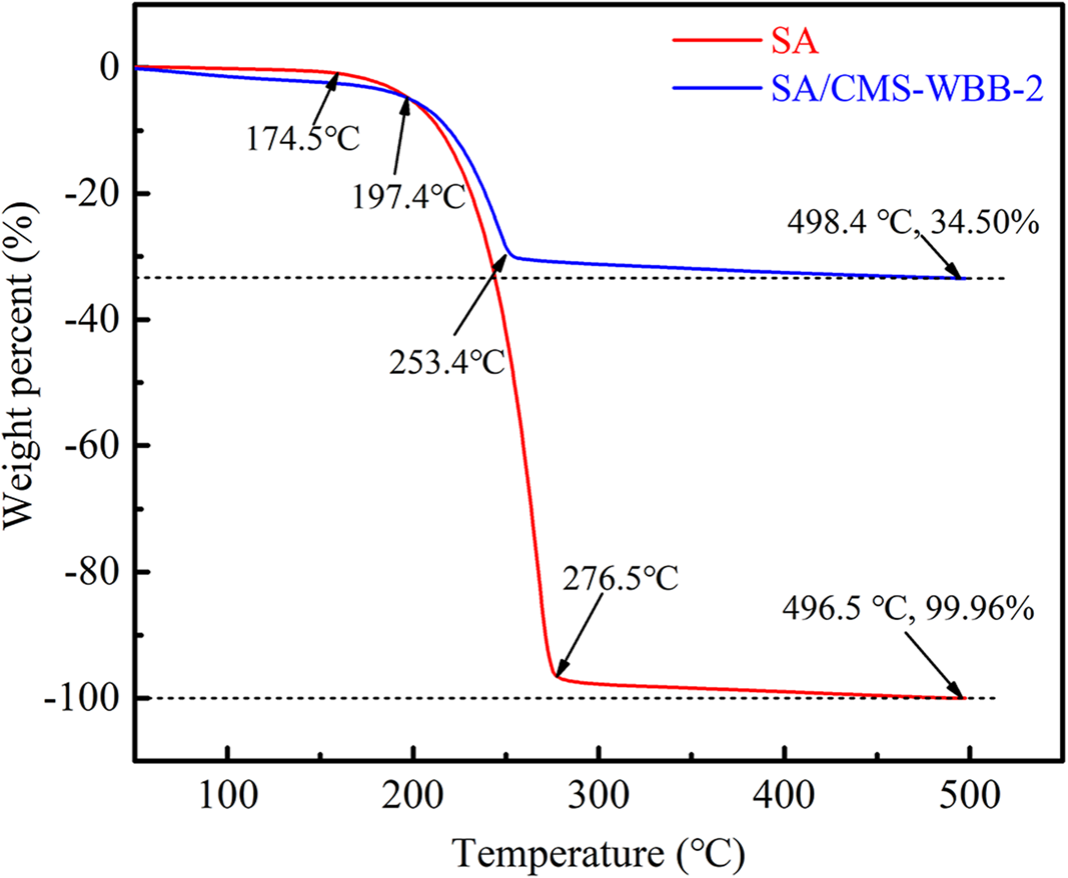
TGA curves of SA and SA/CMS-WBB-2.

The thermal stabilities of pure SA and SA/CMS-WBB-2 were analyzed by TGA, and the results are presented in Fig. 9. The mass loss rate of pure stearic acid was 99.96%. There was a slight weight loss of stearic acid in the temperature range of 20–174.5 °C, which might be owing to the evaporation of water adsorbed on the surface of stearic acid. When the temperature exceeded 174.5 °C, stearic acid began to decompose rapidly, and it almost decomposed completely at 276.5 °C. At the end of the test, there was only 0.04% residue, which might be due to the impurities in stearic acid. SA/CMS-WBB-2 had a slow weight loss in the temperature range of 20–197.4 °C. When the temperature exceeded 197.4v, the SA/CMS-WBB-2 decomposed rapidly, and the weight loss rate slowed down at 253.4v. Finally, the weight loss rate of SA/CMS-WBB-2 was 34.50%. By comparing the curves of SA and SA/CMS-WBB-2, it showed that the shape-stable PCMs of SA/CMS-WBB-2 had favorable thermal stability and high-temperature resistance.

### Latent heat storage analysis of SA and SA/CMS-WBB

**Figure 10:**
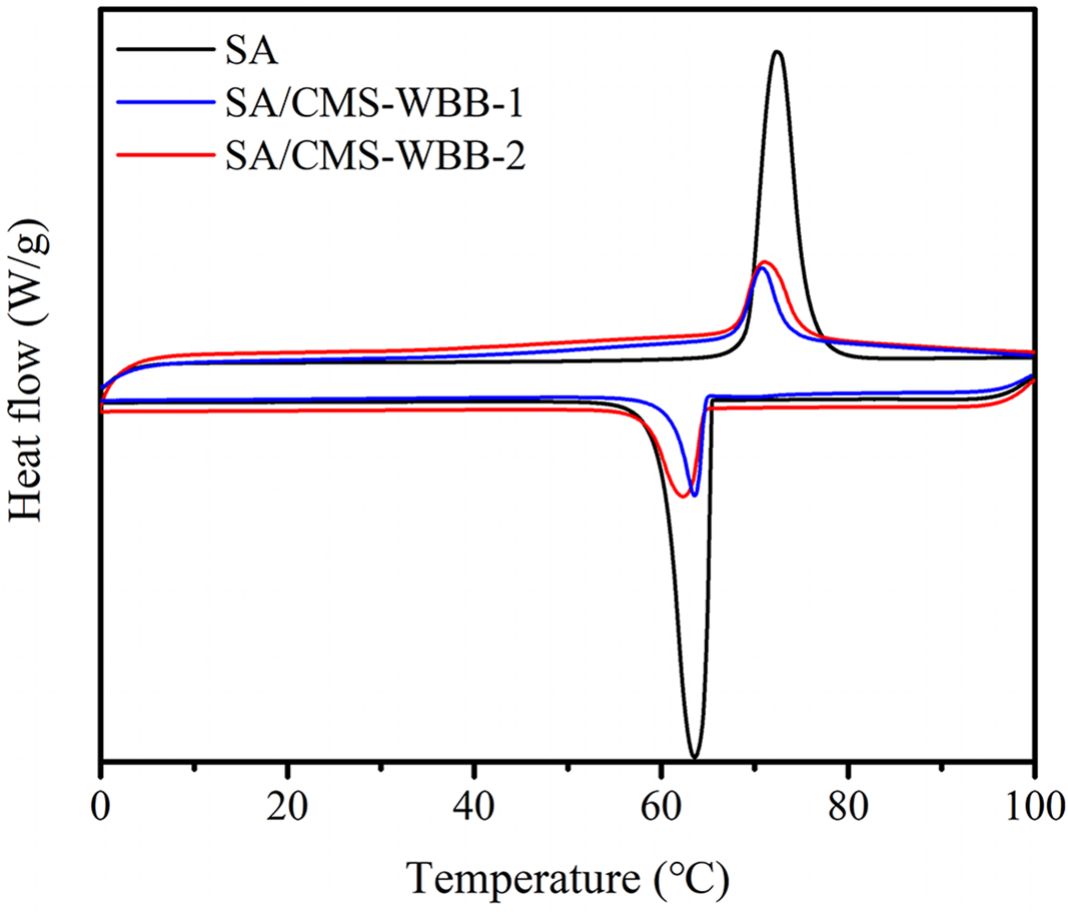
The DSC patterns of SA and SA/CMS-WBB.

**Figure 11:**
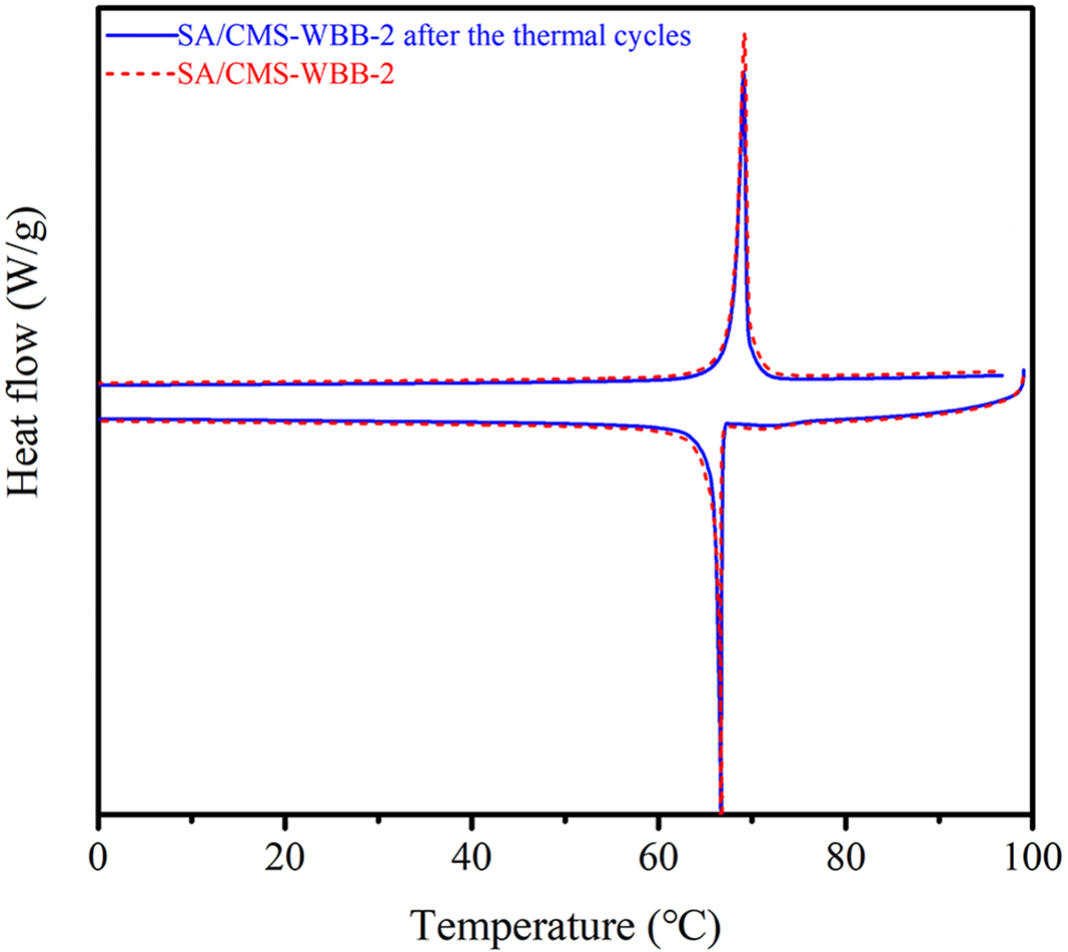
DSC patterns of SA/CMS-WBB-2 before and after thermal cycles.

The DSC curves of SA, SA/WBB and SA/CMS-WBB are presented in Fig. 10, and the DSC thermograms of the composite PCMs and pure stearic acid were similar, which meant SA mainly used as the energy storage material during the phase transition process. The thermal performance parameters of each sample were summarized in Table 1. The melting and crystallization temperature of pure stearic acid were 69.3 °C and 65.4 °C, respectively, and the corresponding values of SA/CMS-WBB-2 were 68.2 °C and 64.5 °C. The results showed that CMS-WBB had little effect on the melting and crystallization temperature of stearic acid in the composite of SA/CMS-WBB. The latent heat of melting and crystallization of stearic acid were 254.2 J/g and 254.5 J/g, and the corresponding values of SA/CMS-WBB-2 were 84.91 J/g and 67.71 J/g, and this was consistent with the DSC patterns in Fig. 10, in which it was observed that the peak area of SA/CMS-WBB composites was less than that of the pure stearic acid. Moreover, the enthalpy of SA/CMS-WBB decreased with the decrease of SA content in the ss-PCMs. The thermal reliability of SA/CMS-WBB-2 was evaluated by 50 thermal cycle tests, and the DSC thermograms of SA/CMS-WBB-2 before and after thermal cycles were displayed in Fig. 11. The changes about the phase transition temperature and phase transition enthalpy of SA/CMS-WBB-2 were negligible before and after the thermal cycle tests, which showed that the SA/CMS-WBB-2 had excellent thermal reliability and great potential application prospects.

**Table 1:** Phase change characteristics of the SA and the prepared samples.

Samples	Melting process			Solidification process		
	Tm (°C)	Tmp (°C)	ΔHm (J/g)	Ts (°C)	Tsp (°C)	ΔHs (J/g)
SA	69.3	72.3	254.2	65.4	63.6	254.5
SA/CMS-WBB-1	68.1	70.7	56.96	64.7	63.6	48.67
SA/CMS-WBB-2	68.2	71.1	84.91	64.5	62.4	67.71

### Thermal conductivity of SA, SA/WBB, and SA/CMS-WBB

**Figure 12:**
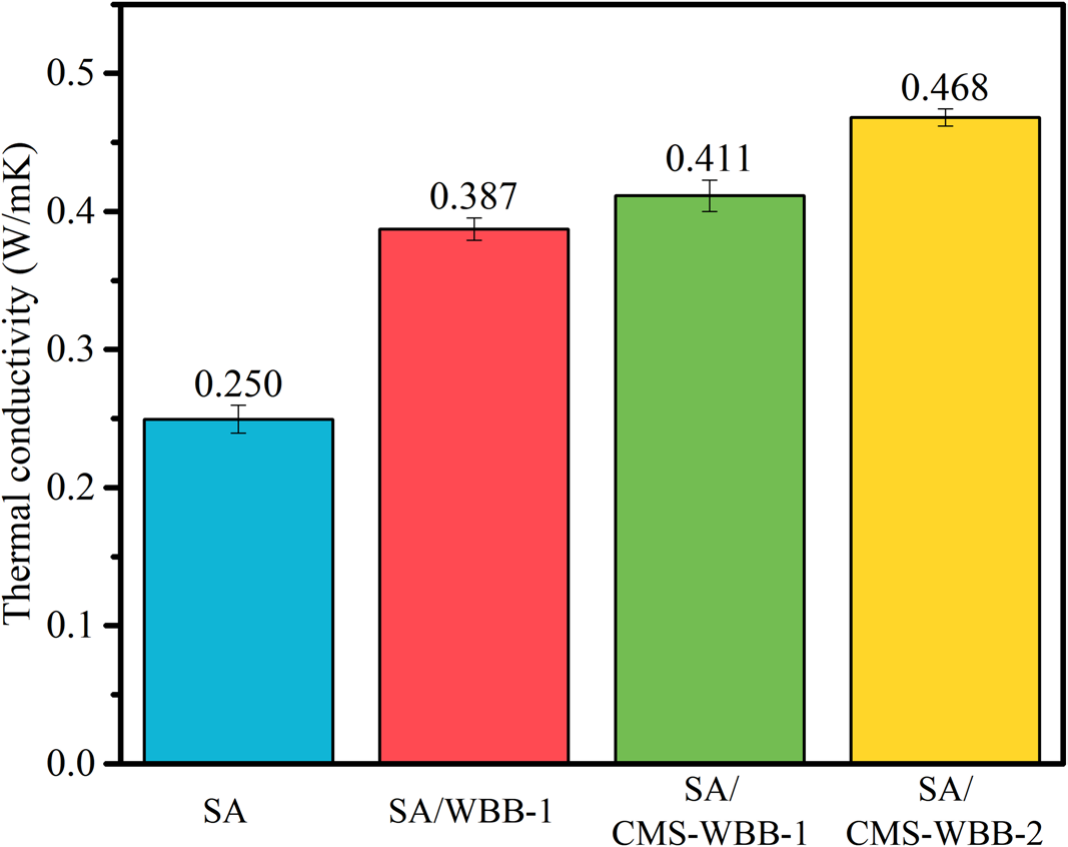
Thermal conductivity of SA, SA/WBB, and SA/CMS-WBB.

In this paper, the thermal conductivity of each sample was investigated, and the results are displayed in Fig. 12. By comparison, when SA was fixed on the carrier, the thermavl conductivity of pure SA was lower than that of the composite PCM, which showed that the strategy of immobilizing SA on the carbon-based carriers to improve the thermal conductivity was effective. Among the samples shown in Fig. 12, the sample of SA/CMS-WBB-2 exhibited the highest thermal conductivity of 0.468 W/mK, and the thermal conductivity of SA/WBB-1 was 0.387 W/mK. Therefore, the strategy of immobilizing SA on the carbon-based carriers to improve the thermal conductivity was effective. Moreover, compared with the thermal conductivity of ss-PCMs prepared bvy using porous carbon as the matrix, such as lauric acid/activated carbon (0.308 W/mK)^31^, biomass succulents carbon/paraffin (0.427 W/mK)^47^, stearic acid/carbonized sunflower straw (0.33 W/mK)^48^, palmitic acid/pinecone biochar (0.3926 W/mK)^17^, almond shell biochar/polyethylene glycol (0.402 W/mK)^16^, LA/PDCWs: lauric acid/porous deep carbonized woods (0.27 W/mK)^24^, the SA/CMS-WBB-2 was competitive, and furthermore, the raw material of wheat bran used in this study was agricultural waste, which could not only realize the reutilization of agricultural waste, but also has the merits of cheap, abundant and easy to obtain. More importantly, the copper ions were adsorbed on the wheat bran powder and copper microspheres were synthesized by one-step in-situ reduction method, which could fix the copper microspheres firmly on the surface of wheat bran biochar and improve the dispersion stability of copper microspheres in SA/CMS-WBB. Hence, the SA/CMS-WBB-2 synthesized in this paper possessed great practical application prospects.

## Conclusion

In this paper, copper microspheres doping wheat bran biochar of CMS-WBB was prepared by a simple one-step pyrolysis method, and the new ss-PCM of SA/CMS-WBB was fabricated by using CMS-WBB as the carrier of SA. The study conclusions indicated that the phase transition points of SA/CMS-WBB-2 were 71.1 °C and 62.4 °C, respectively, and the enthalpy of melting and freezing were 84.91 J/g and 67.71 J/g, respectively. The thermal conductivity of SA/CMS-WBB-2 was 0.468 W/mK, which was raised by 87.2% compared to that of pure stearic acid. In addition, SA and CMS-WBB had favorable chemical compatibility, and the SA/CMS-WBB-2 possessed excellent thermal stability. Therefore, the SA/CMS-WBB developed in this study could be applied as a promising energy storage material in practical utilization.
